# Ergogenic effect of pre-exercise chicken broth ingestion on a high-intensity cycling time-trial

**DOI:** 10.1186/s12970-021-00408-6

**Published:** 2021-02-15

**Authors:** Silvia Barbaresi, Laura Blancquaert, Zoran Nikolovski, Sarah de Jager, Mathew Wilson, Inge Everaert, Siegrid De Baere, Siska Croubels, Stefaan De Smet, N. Tim Cable, Wim Derave

**Affiliations:** 1 Department of Movement and Sports Sciences, Ghent University, Watersportlaan 2, B-9000 Ghent, Belgium; 2 Faculty of Kinesiology, University of Split, Split, Croatia; 3 Institute of Sport, Exercise and Health (ISEH), University College London, London, UK; 4 Department of Pharmacology, Toxicology and Biochemistry, Faculty of Veterinary Medicine, Ghent University, Merelbeke, Belgium; 5 Laboratory for Animal Nutrition and Animal Product Quality, Ghent University, Ghent, Belgium; 6 School of Sport, Exercise and Rehabilitation Sciences, University of Birmingham, Birmingham, UK

**Keywords:** Carnosine, Anserine, Chicken broth, Pre-exercise meal, High-intensity exercise, Performance

## Abstract

**Background:**

chicken meat extract is a popular functional food in Asia. It is rich in the bioactive compounds carnosine and anserine, two histidine-containing dipeptides (HCD). Studies suggest that acute pre-exercise ingestion of chicken extracts has important applications towards exercise performance and fatigue control, but the evidence is equivocal. This study aimed to evaluate the ergogenic potential of the pre-exercise ingestion of a homemade chicken broth (CB) vs a placebo soup on a short-lasting, high-intensity cycling exercise.

**Methods:**

fourteen men participated in this double-blind, placebo-controlled, crossover intervention study. Subjects ingested either CB, thereby receiving 46.4 mg/kg body weight of HCD, or a placebo soup (similar in taste without HCD) 40 min before an 8 min cycling time trial (TT) was performed. Venous blood samples were collected at arrival (fasted), before exercise and at 5 min recovery. Plasma HCD were measured with UPLC-MS/MS and glutathione (in red blood cells) was measured through HPLC. Capillary blood samples were collected at different timepoints before and after exercise.

**Results:**

a significant improvement (*p* = 0.033; 5.2%) of the 8 min TT mean power was observed after CB supplementation compared to placebo. Post-exercise plasma carnosine (*p* <  0.05) and anserine (*p* <  0.001) was significantly increased after CB supplementation and not following placebo. No significant effect of CB supplementation was observed either on blood glutathione levels, nor on capillary blood analysis.

**Conclusions:**

oral CB supplementation improved the 8 min TT performance albeit it did not affect the acid-base balance or oxidative status parameters. Further research should unravel the potential role and mechanisms of HCD, present in CB, in this ergogenic approach.

## Background

Chicken meat and its extracts have long been recognized as a source of bioactive molecules that can potentially improve health status in general, and exercise performance in particular. In traditional South-Asian medicine, chicken extracts are used to alleviate stress or mild disease symptoms [1]. The activities of chicken extracts are suggested to be related to its active components, including proteins, free amino acids, taurine, many minerals, trace elements, and vitamins. In addition, chicken meat extracts contain high concentrations of carnosine (β-alanyl-L-histidine) and anserine (β-alanyl-π-methyl-histidine) in a 1:2 to 1:3 ratio [2, 3].

Carnosine and its methylated analogue anserine are histidine-containing dipeptides (HCD) present in high concentrations in skeletal muscle of mammals and therefore abundantly present in the daily food of non-vegetarian persons [2]. Harris et al. [4] demonstrated that long-term administration of β-alanine - one of the constituent amino acids - is able to raise muscle carnosine concentrations, which leads to enhanced high-intensity exercise performance [5, 6]. The chronic use of chicken breast extract, containing carnosine and anserine, is shown to enhance muscle carnosine levels in humans [7] and to improve high-intensity endurance performance by attenuation of muscle fatigue [8], demonstrating the same mechanism of action of the β-alanine supplementation approach.

In contrast to chronic supplementation strategies, aiming to promote muscle carnosine loading (thus increasing the intracellular muscle pH-buffer capacity), it can be hypothesized that acute supplementation, leading to elevated circulating HCD, may exert ergogenic effects through attenuation of acidosis and/or oxidative stress in blood. Unfortunately, the bioavailability of carnosine in the circulation following supplementation is very low in humans due to the fast breakdown by the very active human serum carnosinase-1 (CN1) enzyme, and acute carnosine supplementation (20 mg/kg BW) could not elicit improvements in a high-intensity cycling test [9]. Interestingly, both in vitro [3, 10, 11] and in vivo [12, 13] experiments revealed that anserine is much less prone to hydrolysis by carnosinase due to the lower affinity to the CN1 enzyme, suggesting that anserine or a combination of anserine plus carnosine might be required to induce acute ergogenic effects. A recent study of Blancquaert et al. (unpublished, congress presentation: http://hdl.handle.net/1854/LU-8642579) demonstrated increased HCD stability in the circulation by a combination of pure (chemically synthesized) carnosine and anserine ingestion in a 1:1 ratio (25 mg/kg BW of each) compared to anserine alone (25 mg/kg BW). In addition, performance-enhancing effects of this pre-exercise supplementation strategy were found on maximal Wingate power in healthy humans.

The ergogenic potential of acute pre-exercise supplementation of chicken extracts (containing both carnosine and anserine) is already tested by Suzuki and colleagues, but the results are so far inconclusive. Suzuki et al. [14] showed that the pre-exercise intake of the chicken breast extract (denominated as CBEX™), containing 0.4 g carnosine and 1.1 g anserine, enhanced the power output of the latter half of 2 sets of repeated sprints. In a follow-up study using the same supplementation strategy for a single set of similar repeated sprints, no improvement on performance was found, although a positive effect on exercise-induced acid-base balance was present [15].

Informed by this emerging evidence for an acute effect of pre-exercise supplementation of pure HCD on exercise performance, we evaluated the effect of a similar dose of HCD in a functional food approach, i.e. through a home-prepared chicken broth (CB) vs a placebo soup (PLA), in a cross-over nutritional intervention study. The purpose of this study is to examine the ergogenic effect of the chicken broth (CB), according to the recipe from Harris et al. [4], administered as a 40-min pre-exercise snack, on a short-lasting, high-intensity cycling exercise (8 min cycling time-trial). The type and duration of the exercise were aimed to challenge the possible ergogenic effects of anserine and carnosine in the CB based on their contribution to the intracellular acid-base balance as well as oxidative stress control [16, 17].

## Methods

### Study design

**Fig. 1:**
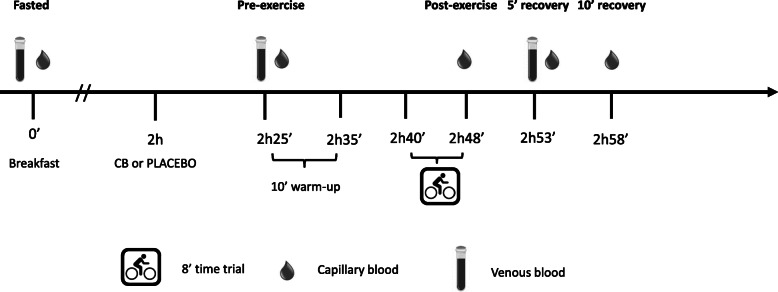
Time schedule of the actual test days (visit 4 and 5)

Twenty healthy, recreationally-trained men voluntarily participated in this double-blind, placebo-controlled, crossover, nutritional intervention study. The participants attended the Aspire Academy Sport Science Laboratory (Doha, Qatar) on five separate occasions. During the first session a maximal incremental oxygen uptake test (VO_2_max) was performed on a cycling ergometer. The second and third visits were reserved for familiarization of the cycling time trial (TT) protocol, consisting of 10 min warm up and 8 min TT. Supplementation conditions (CB or placebo soup) were performed once each, in a random order, on visit 4 and 5 with 1 week time in between. Allocation to the CB and placebo condition was done by an independent researchers, thereby guaranteeing that both subjects and investigators were blinded for the condition. Figure 1 illustrates the time schedule of visits four and five.

### Subjects

Participants underwent a routine medical assessment by a medical doctor and an evaluation of their body mass index (BMI), age and lifestyle was done in the laboratory. A questionnaire was asked for the training rate, food habits, sleeping hours and lifestyle habits. Subject were used to train ~ 10 h/week on average (mixed: swimming-cycling-running-strength). Before the start of the study, a training log was taken and we performed a direct assessment of training status via VO_2_max test. The definition “recreationally-trained” was given after evaluation of the VO_2_max test results (< 55 mL/kg/min, except for 2 participants who were above that value). A food log was required to any participant for the whole duration of the study. Subjects were instructed to maintain their normal dietary habits except for the last meal before the test days where a vegetarian meal was proposed as the only alternative, not to bias the upcoming results on carnosine, anserine on the following day.

Inclusion criteria were males, non-vegetarian, not taking any drugs and/or food supplements 3 month prior and during the study period, medium-high weekly training habits. During the course of the study, 6 subjects dropped out (2 got sick, one relocated to another Country, one for an ankle injury, 2 for work reasons). Of the fourteen subjects that completed the study, subjects’ age, weight and relative maximal oxygen consumption were 37.2 ± 6.5 years, 76.2 ± 10.0 kg and 50.0 ± 5.5 mL/min/kg, respectively. They gave their written informed consent and the Local Ethical Committee (Anti-Doping Laboratory Qatar, ADLQ) approved the study.

### VO_2_max test

The maximal incremental oxygen uptake test (VO_2_max) was performed on a cycling ergometer (SRM bike; Schoberer Rad Messtechnik, Germany) using the Oxycon Pro ergospirometry testing device (Cardinal Health Germany 234 GmbH Leibnizstrasse 7, Hoechberg, Germany). Throughout this cycling test, starting at 100 W for 2 min and with an incremental rate of 50 W/2 min, the subjects maintained a constant pedal cadence of 80 rpm until volitional exhaustion. Maximal oxygen uptake (VO_2_max) was identified through breath-by-breath gas exchange analysis.

### Supplementation

Subjects were instructed to follow a vegetarian meal (thus free of HCDs) on the evening before the test day and to observe 12 h fasting before their arrival. Once in the laboratory, participants were questioned about the compliance of the dinner meal instruction and once assessed it, they received a HCD-free pre-trial meal, designed with standardized nutritional recommendations to help optimize performance during short-term, high intensity exercise (1.5 g/kg BW carbohydrate intake: white bread, jam, Nutella, butter and Gatorade). They ate their breakfast in a separate room. Two hours after their breakfast and 25 min prior to warm-up, subjects consumed 8 mL/kg BW of CB, thereby administering 46.4 mg/kg BW of HCD, or placebo soup similar in taste but without HCD (vegan soup, flavoured with chicken flavour stock powder from InaPaarman’s). CB intake was scheduled 25 min before the start of the exercise sequence (warm up + time trial) based on Everaert et al. [12] who found peak plasma anserine concentrations between 24 and 31 min following intake of nutritionally relevant doses of synthesized anserine. Subjects were allowed to drink water ad libitum throughout the experiment. One person accidentally received CB on both occasions, as clearly appeared from the plasma HCD analysis. This subject was therefore excluded from all analysis, resulting in a trial group of 13 subjects.

### Time trial

The test was performed using the same SRM cycling ergometer as in the maximal incremental oxygen uptake test. The 10 min warm-up for the 8 min TT consisted of the following sequence: 3 min at 100 W, 3 min at 150 W, 3 min at 200 W, 15 s at 60 W, 15 s at 350 W sprint, 15 s at 60 W, 15 s at 350 W sprint. Following 5 min of rest, the 8 min TT was started, during which subjects were free to choose cadence and speed. Subjects received feedback only on the time using a stopwatch, whilst power and cadence were blind during the TT. Bike was set in a linear mode and the mean power during 8 min (expressed as W/kg) was the primary performance outcome measure. Percentage change between the two conditions was calculated. The performance outcome was checked for order effects. Overall, 7 subjects performed best on the first testday, 5 subjects performed best on the second testday, and 1 subject performed equally well on both testdays, assuring that no order effects were present.

### Preparation of CB and placebo soup

The experimental chicken broth was prepared according to Harris et al. [4] with minor modifications. Fresh chicken breast meat was retrieved from a local international food store (Doha, Qatar), with the meat originating from The Netherlands (Europe). Chicken breast (skinned and boned) was finely chopped and boiled for 25 min with water (1 l for every 1.5 kg of chicken). Residual chicken meat was removed by course filtration. The filtrate was flavoured by the addition of carrot, onion, celery, salt, pepper, basil, parsley and tomato puree, and re-boiled for a further 20 min and then cooled before final filtration through fine muslin at 4 °C. The yield from 1.5 kg chicken in 1 l of water was 870 mL of stock. Placebo soup was prepared using the same flavouring agents and vegetables in boiling water, without adding any meat. Single portions of both CB and placebo soup were prepared according to any participant’s body weight (8 mL mL/kg BW) and frozen in plastic bottles at − 80 °C until consumption. Each bottle was placed in a closed freezing bag for hygienic conditions. Only the freezing bags containing the bottles were labelled in order to guarantee double-blinding during the test days.

### HCD in CB and placebo soup

Carnosine and anserine levels were quantified in both CB and placebo soup preparations through HPLC-fluorescence on two representative samples of each soup, following the method described by Everaert et al. [18]. The carnosine and anserine concentrations of the CB were 8.61 and 16.05 mmol/L respectively and found below the limit of detection in the placebo soup. The subjects thus consumed 1181 ± 156 mg carnosine (15.6 mg/kg BW), 2340 ± 308 mg anserine (30.8 mg/kg BW), totalling 3522 ± 464 mg of HCD (46.4 mg/kg BW) in a portion of 607 ± 80 mL chicken broth (8 mL/kg BW).

### Blood gas parameters

Capillary blood samples (70 μL) were taken by finger pricking and analysed with a blood gas analyzer (ABL90 Flex; Radiometer, Brønshøj, Denmark) at five different time points: fasted (at arrival), before warm-up, following 8 min TT, after 5 min and 10 min recovery. The samples were analysed for glucose, pH, lactate, bicarbonate and electrolytes (K^+^, Ca^2+^, Cl^−^ and Na^+^).

### Plasma HCD levels

Venous blood samples (EDTA) were collected at three time points: fasted, pre-exercise and 5 min post-exercise recovery. Precooled (4 °C) EDTA tubes were centrifuged immediately after blood collection at 4 °C to separate plasma. Plasma samples were deproteinized with 35% sulfosalicylic acid (SSA) and stored immediately at − 20 °C until analysis. These samples were analysed for plasma carnosine and anserine by an in-house developed and validated UPLC-MS/MS method. The analytical standards of L-carnosine and L-anserine were chemically synthesized by Flamma S.p.a. (Chignolo d’Isola, Bergamo, Italy). The internal standard (IS), carnosine-D4, was purchased from Sanbio B.V. (Uden, The Netherlands). Acetonitrile (ACN), methanol and formic acid (FA) were ULC-MS grade and obtained from Biosolve (Valkenswaard, The Netherlands). A Milli-Q® water system (Merck Millipore, Darmstadt, Germany) was used to obtain ultrapure water. Deproteinized plasma was vortexed and 150 μL was added to 240 μL of methanol containing 1% formic acid and 10 μL IS (2.5 μM in water). Samples were vortexed (30 s) before centrifugation (15 min, 4 °C, 15000 g) and 350 μL of supernatant was evaporated (35 °C, vacuum, Gyrovap). The remaining droplet of about 20 μL was redissolved in 90 μL ultrapure water and vortexed for 30 s before being transferred to an autosampler vial (Filterservice, Eupen, Belgium). A 5 μL aliquot was injected onto the UPLC-MS/MS system.

Analyses were performed on an UPLC-MS/MS platform consisting of an Acquity H-Class Quaternary Solvent Manager and Flow-Through-Needle Sample Manager with temperature controlled tray (8 °C) and column oven (45 °C), all from Waters (Milford, MA, USA). Chromatographic separation was achieved on an Acquity UPLC HSS T3 column (100 × 2.1 mm, dp: 1.8 μm, Waters) in combination with an Acquity HSS T3 1.8 μm Vanguard pre-column, both from Waters. Gradient elution was established with a mobile phase consisting of 0.1% (v/v) FA in water (solvent A) and 0.1% (v/v) FA in ACN (solvent B) at a flow rate of 0.4 mL/min. The following gradient was used: 0.0–1.5 min (99% A, 1% B), 1.5–2.0 min (linear gradient to 5% A, 95% B), 2.0–3.5 min (5% A, 95% B), 3.5–4.0 min (linear gradient to 99% A, 1% B), 4.0–7.0 min (99% A, 1% B). The UPLC column effluent was interfaced to a Xevo TQ-XS® MS/MS system, equipped with an electrospray ionization (ESI) probe operating in the positive mode (all from Waters). A divert valve was used and the UPLC effluent was directed to the mass spectrometer from 0.2 to 3.0 min. Instrument parameters were optimised by direct infusion of working solutions of 100 ng/mL of carnosine, anserine and the IS, respectively, at a flow-rate of 10 μL/min and in combination with the mobile phase (50% A, 50% B, flow-rate: 200 μL/min). The settings on the Xevo TQ-XS® were as follows: desolvation gas flow rate: 800 L/h; desolvation temperature: 500 °C; cone gas flow rate: 150 L/h; source temperature: 150 °C. The capillary voltage was optimized at 3.00 kV for ESI in positive ionization mode. The detector was operating in multiple reacting monitoring mode, scanning the two most intense transitions of carnosine: *m/z* 227.2 > 110.1 (quantification ion, cone = 35 V; collision energy (CE) = 20 eV) and *m/z* 227.2 > 156.1 (confirmation ion, cone = 35 V; CE = 13 eV), anserine: *m/z* 241.2 > 109.1 (quantification ion, cone = 30 V; CE = 23 eV) and *m/z* 241.2 > 170.1 (confirmation ion, cone = 30 V; CE = 15 eV) and carnosine-D4 (IS): *m/z* 231.0 > 110.1 (quantification ion, cone = 30 V; CE = 22 eV) and *m/z* 231.0 > 156.0 (confirmation ion, cone = 30 V; CE = 15 eV). Masslynx software 4.2 (Waters) was used for instrument control and data extraction.

Pooled EDTA plasma used to prepare the calibration curve and quality control samples was obtained from 2 healthy volunteers who followed a lacto-ovo-vegetarian diet free of HCD for 2 days prior to blood withdrawal. This plasma pool contained basal carnosine and anserine concentrations of 23.34 ± 2.78 nmol/L and 7.93 ± 4.40 nmol/L, respectively. For quantification, a 10-point calibration curve was prepared by spiking aliquots of the pooled deproteinized EDTA plasma with known concentrations of carnosine and anserine ranging between 5 and 15,000 nmol/L. Calibration curves showed good linearity (r > 0.99 and goodness-of-fit coefficient < 20%, Table 1). Quality control (QC) was performed by spiking pooled plasma with 50, 500 and 5000 nmol/L of the dipeptides. The results of these QC samples could also be used to evaluate the within-run and between-run precision of the LC-MS/MS method, which fell within the acceptance ranges (Table 2). Calculated limit of detection (LOD) values were 5.72 nmol/L and 10.61 nmol/L for carnosine and anserine, respectively (Table 1). No carry-over of the analytes of interest was observed on the UPLC-MS/MS instrument. Results are expressed as absolute concentrations and the change in carnosine and anserine concentrations in blood due to supplement intake was calculated by subtracting baseline values from the 5 min recovery values.

**Table 1:** Results of the evaluation of linearity (slope (a), intercept (b), goodness-of-fit coefficient (g), correlation coefficient (r)), limit of quantification (LOQ), limit of detection (LOD) for the LC-MS/MS analysis of carnosine (CAR) and anserine (ANS) in human plasma

Component	Calibration Range (nM)	a	b	g (%)	r	LOQ (nM)	LOD^a^ (nM)
CAR	5–10,000	0.035 ± 0.046	0.202 ± 0.017	8.5 ± 0.7	0.9961 ± 0.0006	50	5.7
ANS	5–10,000	0.006 ± 0.000	0.044 ± 0.021	6.0 ± 2.2	0.9978 ± 0.0015	50	10.6

Note: acceptance criteria: *r* ≥ 0.99, g ≤ 20%; ^a^LOD = 3 x S_res_/a with S_res_ = residual standard deviation (corresponding with the standard deviation of the intercept (b) of minimal 3 calibration curves) and a = slope

**Table 2:** Results of the within-run and between-run precision and accuracy evaluation for the analysis of carnosine (CAR) and anserine (ANS) in plasma using LC-MS/MS

Component	Theoretical concentration (nM)	Mean concentration ± SD (nM)	Precision, RSD (%)	Accuracy (%)
CAR	50 ^a^	54.7 ± 2.3	4.2	9.4
	50 ^b^	54.4 ± 2.0	3.7	8.7
	500 ^a^	487.5 ± 20.0	4.1	−2.5
	500 ^b^	491.8 ± 17.1	3.5	−1.6
	5000	4328.1 ± 190.7	4.4	− 13.4
	5000 ^b^	4419.0 ± 257.1	5.8	−11.6
ANS	50 ^a^	53.8 ± 2.4	4.4	7.6
	50 ^b^	53.3 ± 2.4	4.6	6.6
	500 ^a^	483.7 ± 21.2	4.4	−3.3
	500 ^b^	499.1 ± 25.8	5.2	−0.2
	5000	4307.6 ± 116.6	2.7	−13.8
	5000 ^b^	4509.2 ± 322.1	7.1	−9.8

Concentrations of 50, 500 and 500 nM correspond with 11.3, 113.1 and 1131.2 ng mL^− 1^ of CAR and 12.0, 120.1 and 1201.3 ng mL^− 1^ of ANS, respectively; acceptance criteria: accuracy: ≥ 10 to < 100 ng mL^− 1^: − 30 to + 10%, ≥ 100 ng mL^− 1^: − 20 to + 10%, within-run precision (RSD_max_): ≥ 10 to < 100 ng mL^− 1^: 15.0%, ≥ 100 ng mL^− 1^: 10%; between-run precision: ≥ 10 to < 100 ng mL^− 1^: 23%, ≥100 ng mL^− 1^: 16% [19].

Note: ^a^ Within-run accuracy and precision (*n* = 6); ^b^ Between-run accuracy and precision (*n* ≥ 3 per day, at least 3 analysis days); *SD* Standard deviation, *RSD* Relative standard deviation

### Glutathione levels

Heparin venous blood samples to determine glutathione levels were collected at arrival (fasted), before warm-up and following exercise. Heparinized blood was treated with BPDS (bathophenanthrolinedisulfonic acid disodium salt hydrate solution) 1 mM and after centrifugation, 300 μL red blood cells (RBC) were stored at − 20 °C until further analysis. Quantification of reduced and oxidized glutathione forms (GSH and GSSG, respectively) was based on methods previously described by Reed et al. [20] and Yoshida [21]. In short, the derivatization procedure includes the reaction of iodoacetic acid with thiols to form S-carboxymethyl derivatives followed by chromophore derivatization of primary amines with Sanger’s reagent, 2,4-dinitrofluorobenzene at pH 8–9 overnight at 4 °C. Samples were centrifuged, filtered (cellulose syringe filter of 0.20 μm) and transferred to a HPLC vial. Derivatives were separated o*n* a 3-aminopropyl column (EC250/4.6 Nucleosil 120–7 NH2; C18 4.6 × 150 mm, 5 μm) by reversed-phase ion-exchange HPLC (Agilent 1200 series). The separation was carried out at 1.50 mL/min and 40 °C. The eluted derivatives were measured by detection at 365 nm (DAD detector). GSH and GSSG were identified by retention times of authentic internal and external standards.

### Plasma CN1 activity

CN1 activity was determined on heparinized plasma from the fasted state of the placebo supplementation test day, according to the method described by Teufel et al. [22]. Briefly, the reaction was initiated by addition of substrate (L-carnosine) to a heparinized plasma sample and stopped after 10 min of incubation at 37 °C by adding 1% sulfosalicylic acid (SSA). Liberated histidine was derivatized with o-phthaldialdehyde, and the maximum increase was used for determining the maximum activity. Fluorescence was measured by excitation at 360 nm and emission at 460 nm.

### Statistics

A paired T-test was used to evaluate the effect of CB or placebo ingestion on mean power output of the 8 min TT. A 2 × 3 repeated measures analysis of variance ANOVA was used to evaluate plasma carnosine and anserine levels and glutathione levels in RBC with condition (CB or placebo) and time (fasted, pre-exercise and 5 min recovery) as within factors. A 2 × 5 repeated-measures ANOVA was used to evaluate glucose, pH, lactate, bicarbonate and electrolytes with condition (CB or placebo) and time (fasted, pre-exercise, post-exercise, 5 min recovery, 10 min recovery) as within factors. In case of a significant interaction, a Tukey post hoc analysis was performed. In case of missing values, a mixed-effects analysis was used. Correlations between CN1 activity and performance output were obtained by means of Pearson correlations. All statistical analyses were performed with Graphpad Prism version 8.0. Values are presented as mean ± SD and statistical significance threshold was set at *p* ≤ 0.05.

## Results

### Exercise performance

**Fig. 2:**
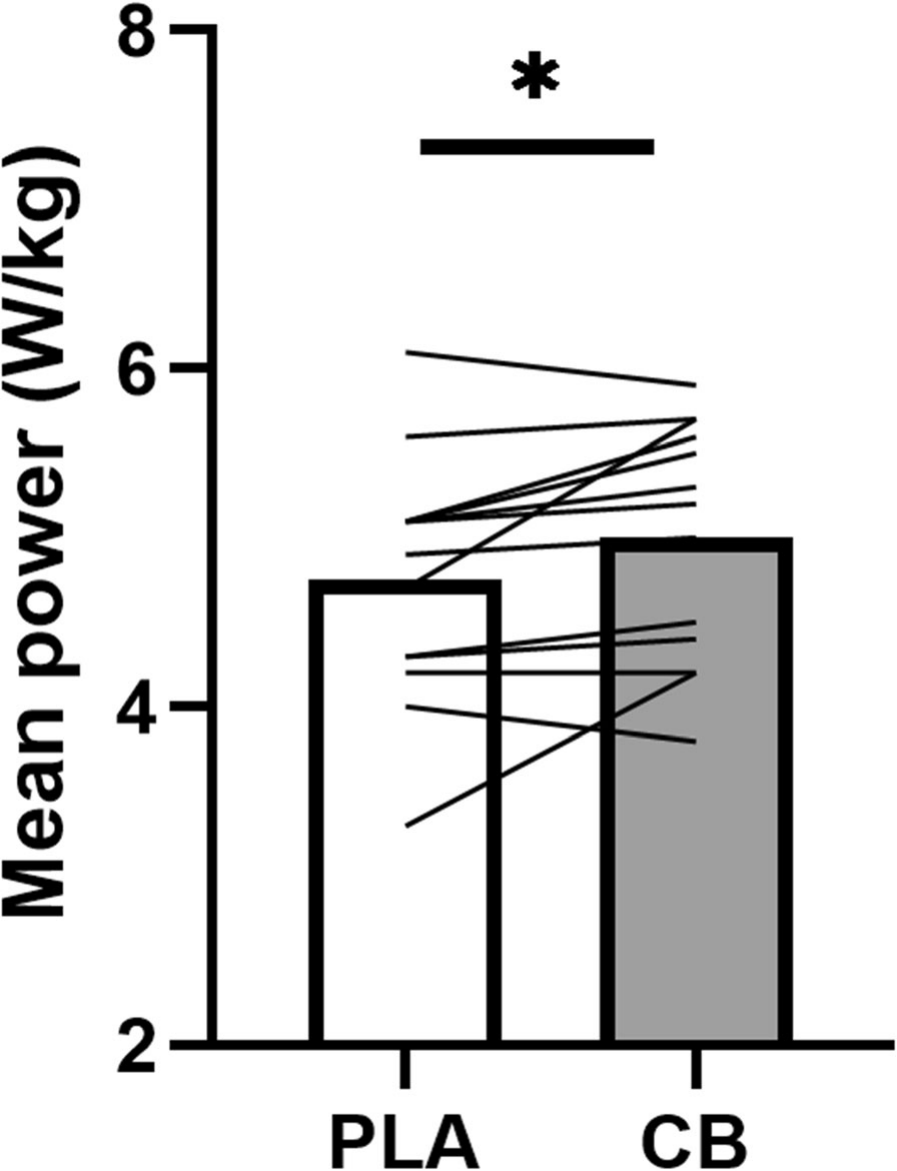
Mean relative power output (Watt/kg) of the 8 min TT following Placebo (PLA) or Chicken Broth (CB) supplementation. Bars represent the mean, lines represent individual values (*N* = 13). * *p* = 0.033

A significant difference (*p* = 0.033, t = 2.406) was observed between the CB and placebo (PLA) supplemented conditions for mean power during the 8 min TT (Fig. 2). Mean relative power output was 5.2% higher following CB supplementation compared to placebo (5.0 W/kg ± 0.7 vs 4.75 W/kg ± 0.7, respectively).

### Plasma HCD values

**Fig. 3:**
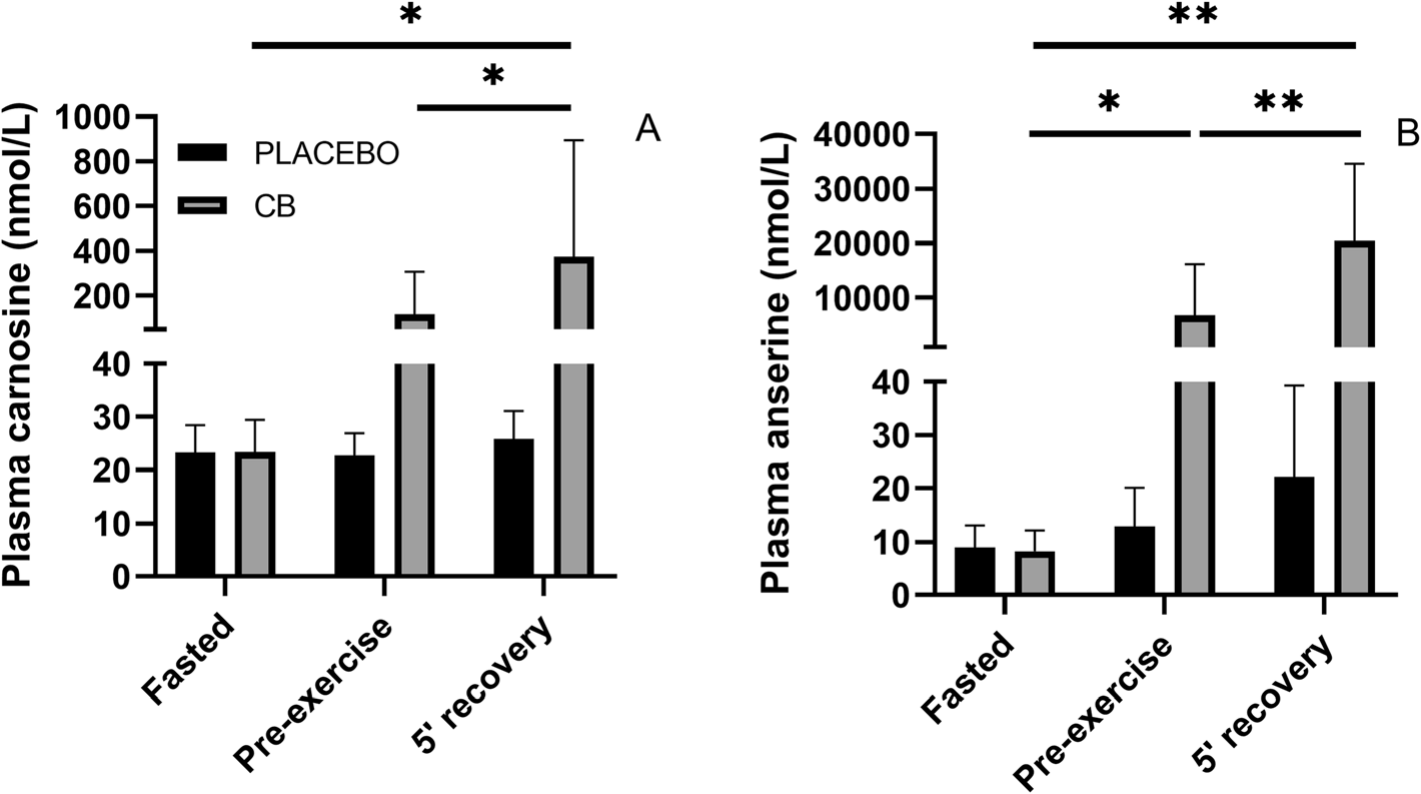
Plasma carnosine (**a**) and anserine (**b**) concentrations following either Placebo or Chicken Broth (CB) supplementation. Samples were collected in a fasted state, pre-exercise (25 min after supplementation) and after 5 min recovery post-exercise (53 min after supplementation). Values are means ± SD (*N* = 13); * *p* < 0.05; ** *p* < 0.001 significant difference between time points

The results of the in-house validation of the LC-MS/MS method that was used to quantitate carnosine and anserine in plasma are shown in Table 1 (linearity, limit of quantification or LOQ and limit of dectection or LOD) and Table 2 (within-run and between-run accuracy and precision), respectively. As can be seen, all results fell within the acceptance ranges as recommended by international guidelines [19], indicating that the method was fit-for-purpose. Figure 3 shows the concentrations of plasma carnosine (A) and anserine (B) both in a fasted state, pre-exercise (25 min after supplementation) and after 5 min recovery post-exercise (53 min after supplementation), following either placebo or CB supplementation. Both plasma carnosine and anserine demonstrate low concentrations in the nanomolar range in a fasted state (range plasma carnosine: 17–30 nmol/L; range plasma anserine: 2–17 nmol/L). A significant interaction effect was present for both plasma carnosine (*p* = 0.011, F = 5.573) and anserine (*p* <  0.001, F = 21.48). Plasma carnosine and anserine did not significantly change over time following placebo supplementation, while both metabolites are gradually and highly elevated following CB ingestion. Significantly higher plasma carnosine and anserine levels are found at 5 min recovery post-exercise (53 min after supplementation) compared to fasted samples and pre-exercise samples (25 min after supplementation). Interestingly, plasma anserine at 5 min recovery post-exercise is 50 times higher compared to plasma carnosine (20,424.7 nmol/L vs 372.7 nmol/L, respectively).

### Correlations

A significant negative correlation (*r* = − 0.692; *p* = 0.011) was observed between the CN1 activity and the change in plasma anserine from the fasted to 5 min recovery time point (delta) in the CB supplementation condition, demonstrating that higher CN1 activity attenuates accumulation of plasma anserine following CB ingestion. No significant correlations were found between CN1 activity and change in plasma carnosine at these time points (PLA: *r* = − 0.333; *p* = 0.266; CB: *r* = − 0.311; *p* = 0.302). However, following CB supplementation, the change in plasma carnosine was significantly correlated to the change in plasma anserine (*r* = 0.790; *p* = 0.001), demonstrating that the accumulation of both compounds in plasma is interrelated.

No significant correlation between CN1 activity and percent improvement in mean power (CB vs. placebo) was seen (*r* = 0.237; *p* = 0.436). Furthermore, no significant correlation (*r* = − 0.102, *p* = 0.740) was found between the change in plasma anserine levels and percent improvement in mean power (CB vs placebo).

### Glutathione status

**Fig. 4:**
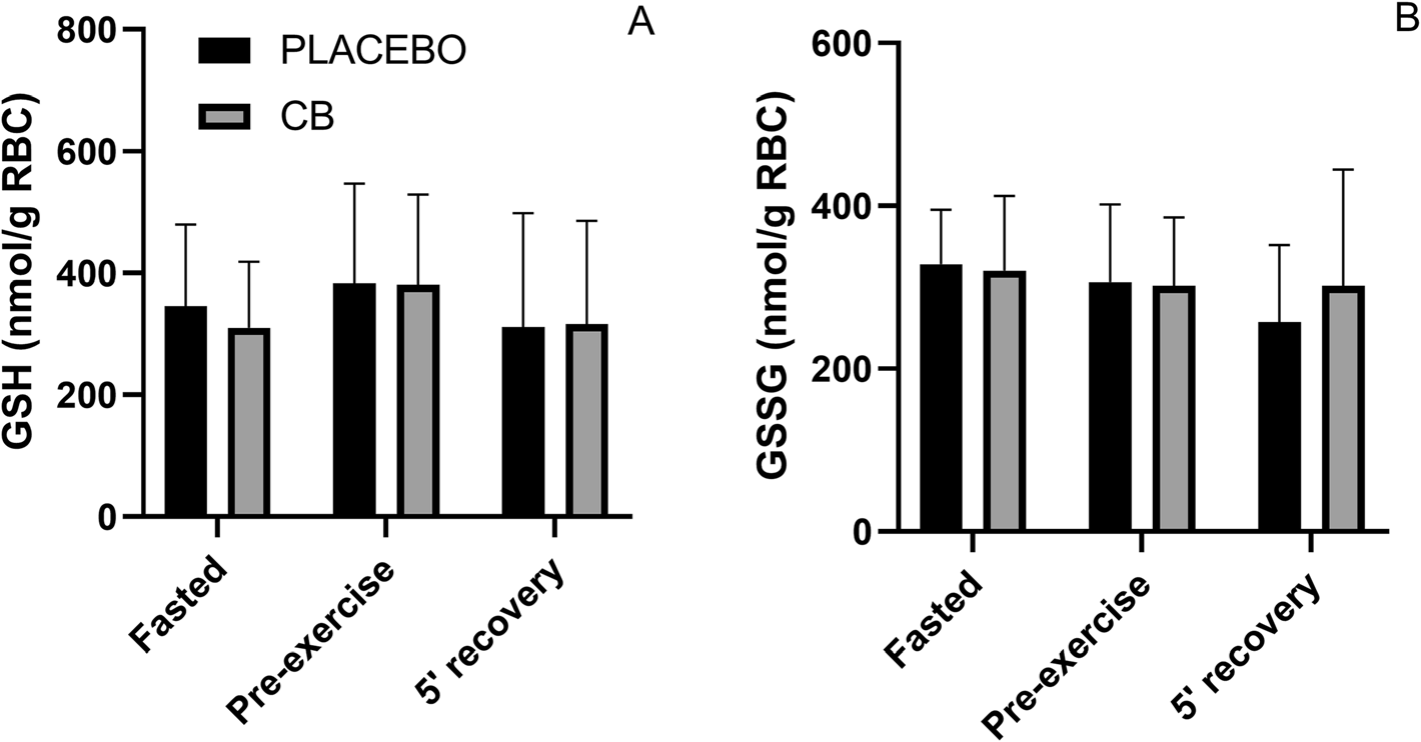
Reduced glutathione (GSH) (**a**) and oxidized glutathione (GSSG) (**b**) values in red blood cells for placebo and Chicken Broth (CB) conditions at three time points. Values are means + SD (*N* = 13)

No significant interaction effect was observed for blood glutathione values (measured in red blood cells (RBC)), neither for the reduced form (GSH, *p* = 0.771, F = 0.2620, Fig. 4a), nor for the oxidized one (glutathione disulphide or GSSG, *p* = 0.267, F = 1358, Fig. 4b). Furthermore, no main effect of time or supplement was present, suggesting glutathione status is not affected by CB supplementation.

### Capillary blood analysis

Among all the parameters covered by the capillary blood analysis (pH, lactate, bicarbonate, glucose and electrolytes such as Ca^2+^, Cl^−^ and Na^+^), no significant interaction effect was observed, except for K^+^ (see Table 3). However, no significant difference was found for K^+^ values between conditions at any of the time points. The difference in pre-exercise to post-exercise K^+^ values demonstrate a slightly higher increase following placebo ingestion compared to CB (1.13 vs 0.55 mmol/L, respectively), but this did not reach statistical significance. Furthermore, all the above-mentioned parameters, except Cl^−^, showed a significant time effect, demonstrating that the exercise affects these parameters independently of condition.

**Table 3:** Capillary blood analysis after acute Placebo or Chicken Broth (CB) supplementation at 5 time points

Capillary blood analysis													
Supplementation (S)	Placebo					CB					***p***-values		
**Time (T)**	fasted	pre-exercise	post-exercise	5 min rec	10 min rec	fasted	pre-exercise	post-exercise	5 min rec	10 min rec	T	S	TxS
**Lactate (mmol/L)**	1.19 (0.47)	2.05 (0.78)*	15.74 (3.91)*	13.81 (2.21)*	11.98 (1.98)*	1.17 (0.56)	2.15 (0.63)*	16.01 (2.53)*	13.84 (3.00)*	11.85 (3.14)*	**< 0.001**	0.837	0.994
**pH**	7.41 (0.020)	7.39 (0.019)	7.18 (0.056)*	7.20 (0.056)*	7.25 (0.046)*	7.41 (0.014)	7.39 (0.022)	7.17 (0.078)*	7.20 (0.078)*	7.26 (0.070)*	**< 0.001**	0.800	0.299
**Bicarbonate (mmol/L)**	25.0 (1.3)	24.4 (1.0)	12.8 (1.6)*	13.4 (1.8)*	14.6 (1.7)*	25.0 (0.9)	24.1 (1.0)	12.7 (1.9)*	13.4 (2.3)*	15.1 (2.5)*	**< 0.001**	0.861	0.136
**Lactate/protons**	0.030 (0.012)	0.050 (0.018)*	0.24 (0.054)*	0.22 (0.022)*	0.21 (0.020)*	0.030 (0.015)	0.053 (0.016)*	0.24 (0.025)*	0.21 (0.021)*	0.21 (0.027)*	**< 0.001**	0.910	0.691
**Glucose (mmol/L)**	5.09 (0.32)	5.96 (1.00)*	6.16 (1.36)*	6.18 (1.06)*	5.75 (0.80)	5.32 (0.56)	5.64 (0.84)	6.48 (1.57)*	6.18 (1.35)	5.68 (1.26)	**0.001**	0.541	0.225
**Potassium (mmol/L)**	4.31 (0.27)	4.17 (0.33)	5.34 (1.2)*	4.00 (0.29)*	4.20 (0.43)	4.23 (0.43)	4.40 (0.40)	5.00 (0.37)*	4.10 (0.30)	4.43 (0.29)	**0.004**	0.972	**0.007**
**Calcium (mmol/L)**	1.14 (0.076)	1.14 (0.062)	1.17 (0.077)	1.12 (0.058)	1.11 (0.055)	1.14 (0.060)	1.12 (0.046)	1.15 (0.071)	1.09 (0.065)	1.10 (0.063)*	**0.025**	0.412	0.959
**Chloride (mmol/L)**	114.1 (3.0)	113.2 (2.9)	114.7 (2.7)	112.2 (1.5)	113.5 (2.4)	114.1 (3.1)	115.0 (2.8)	115.3 (3.6)	114.1 (3.5)	113.5 (2.9)	0.268	0.358	0.272
**Sodium (mmol/L)**	137.5 (2.7)	139.2 (2.1)	142.1 (4.0)*	140.5 (2.8)*	139.2 (4.7)	137.5 (2.4)	138.0 (2.0)	141.8 (2.4)*	139.8 (3.2)	138.8 (2.3)	**0.014**	0.520	0.412

Values are means (SD). Values are significantly different at *p* ≤ 0.05. * significant different from fasted

## Discussion

The present study demonstrates that acute pre-exercise chicken broth (CB) ingestion significantly increased plasma concentrations of both carnosine and anserine. In support of our hypothesis, we report an increase in mean power during an 8-min cycling time-trial, compared to a vegetarian placebo soup. The ergogenic effect of acute pre-exercise chicken breast extract (CBEX) ingestion was previously explored in two Japanese studies, but with conflicting conclusions. While Suzuki et al. [14] showed that the pre-exercise intake of a chicken breast extract enhanced the power output of the latter half of 2 sets of short maximal repeated sprints, their follow-up study could not confirm this [15]. The current study demonstrates a sizeable performance improvement of 5% by pre-exercise chicken broth ingestion in a sports-relevant type of high-intensity exercise.

The main difference between the experimental (CB) and the placebo soup was the presence of extracted chicken meat compounds in the former. Although in theory many molecules could potentially be responsible for the ergogenic effect, there is a body of evidence suggesting that the HCDs, carnosine and anserine, play a pivotal role. First, the HCDs are the most abundant organic compounds in the chicken broth and account for approximately 10% of its dry mass (unpublished data from our lab). More importantly, we recently presented performance-enhancing effects of the pre-exercise ingestion of a mixture of pure (chemically synthesized) carnosine and anserine on maximal Wingate power in humans (Blancquaert et al., congress presentation: http://hdl.handle.net/1854/LU-8642579). Although the type of exercise was not identical, the approach (oral supplementation 30–40 min prior exercise) and dose (40 mg/kg HCD, ratio 1:1 carnosine:anserine) were very similar. It remains to be determined whether the findings from the pure supplement powder approach of Blancquaert et al. and the functional food approach from the current study rely on the same mechanism, i.e. elevating circulating HCD levels prior to intense exercise. Based on the design of our experiment, we can, however, not fully rule out the possible involvement of macro- and micronutrients, other than HCDs, to underly the ergogenic effects found in the current paper.

Upon oral ingestion, carnosine is intactly absorbed as a dipeptide but is then rapidly degraded by serum CN1 into β-alanine and histidine with an elimination half-life of merely 1 or 2 min [23]. In contrast, anserine degradation in blood is less efficient than carnosine, due to the lower affinity to the CN1 enzyme and the anserine half-life in human serum is in the range of 5–20 min [3, 10–12]. In the current study, CB intake promoted a significant increase in both plasma carnosine and anserine in the post-exercise (53 min after supplementation) time point compared to the fasted state. In line with the expectations from the higher CN1 affinity for carnosine compared to anserine, the increase in plasma carnosine was 50 times lower compared to the increases in plasma anserine, whereas the carnosine:anserine ratio in the broth was 1:2. In addition, there was a negative correlation between the CN1 activity and the anserine increase in blood (also called “anserinemia”) after CB intake, demonstrating that a lower CN1 activity promotes more accumulation of plasma anserine.

We have previously shown that elite athletes who excel in short and high-intensity sport disciplines have a lower serum CN1 activity compared to untrained controls and to endurance athletes [9]. Given the low intraindividual variation and the genetic basis for serum CN1 activity, that finding may have been the result of performance-related genetic selection [9]. It could be hypothesized that athletes with a low CN1 activity have higher circulating HCD levels (upon dietary intake of meat or fish) and therefore an ergogenic advantage for subsequent high-intensity exercise performance. In the current study, we found evidence for the former, but not for the latter, as we observed a correlation between CN1 activity and plasma HCD levels, but not with performance improvements. The individual performance data show (Fig. 2) show improvement in eleven subjects, of which two subjects seem to have had a markedly larger improvement. However, this interindividual variation in responsiveness could not be explained by variation in CN1 activity.

In search for a potential ergogenic mechanism, the pH buffering should be explored as the imposed type of brief cycling TT severely challenges the pH (decline from 7.4 to 7.2), bicarbonate (from 25 to 12 mmol/L) and lactate (from 1 to 16 mmol/L) system. The ergogenic mechanism of the muscle carnosine loading strategy by chronic β-alanine supplementation is known to include attenuation of exercise-induced acidosis by improved muscle pH buffering capacity [4, 17]. It was suggested by Suzuki et al. [15] that also acute pre-exercise carnosine/anserine supplementation (through chicken breast extract ingestion) can function as an extracellular pH buffer in the circulation, as they observed a significant sparing of blood bicarbonate during repeated sprints. Also Baguet et al. [9] found a hint for such action, as pre-exercise carnosine ingestion mildly elevated resting bicarbonate levels. Carnosine and anserine have an ideal pKa (6.72 and 7.01 for the imidazole nitrogen of carnosine and anserine, respectively [2]), which is superior to that of bicarbonate to serve as a physiological pH buffer. However, in the current study, clearly no significant effects of CB supplementation were observed on either pH, lactate or bicarbonate. In hindsight, this is not fully surprising. Despite their ideal pKa, it should be acknowledged that the almost 700-fold increase in plasma HCD levels (from 30 nmol/L to 20 μmol/L) attained in this study, does not by far approach the plasma concentrations of bicarbonate, which is still a further 1000-fold higher (25 mmol/L). Also in comparison to the skeletal muscle carnosine concentrations, which are in the millimolar range and can increase by 50% or more in response to chronic β-alanine supplementation, the currently observed elevation of plasma carnosine and anserine is several orders of magnitude lower and likely too low to be physiologically meaningful to affect acid-balance during exercise.

After excluding improved pH buffering as a potential mechanism of action, it could be hypothesized that the other prominent bio-active property of HCDs is involved, namely that of an antioxidant. Acute pre-exercise administration of the antioxidant N-acetyl-cysteine has been shown to improve exercise performance in humans [24]. Also carnosine and anserine have been widely investigated and consensually accepted as powerful antioxidants [2, 25, 26], but it is unclear whether they can be involved in protection against oxidative stress and fatigue during exercise in vivo. Chronic β-alanine supplementation has been shown to reduce a marker of lipid peroxidation (TBARS) during downhill running in rats [27], but data in humans on chronic supplementation of β-alanine [28] or acute supplementation of carnosine [9] did not show reduction in oxidative stress. Recently, one study [29] suggested that acute pre-exercise anserine supplementation (15 mg/kg or 30 mg/kg) could alleviate exercise-induced oxidative stress. However, the methodology of this study is unsubstantial and results are poorly reported, making it hard to rely on these conclusions. In the current study, no significant effect was observed on blood glutathione values in erythrocytes, neither on the reduced (GSH) nor oxidized (GSSG) form, suggesting that the ergogenic effect of the CB is not a result of its antioxidant capacities. As such, the potential ergogenic mechanism underlying the present findings is still unclear and warrant further investigation.

As the nutritional intervention merely involved the ingestion of a carefully prepared bowl of chicken broth, no adverse symptoms were expected. Indeed, we reported no side-effects in any of the subjects in the present study. The ingested HCD dose was twice as high as in the studies of Suzuki et al. [14, 15]. In the study of Everaert et al. [18] a pharmacological dose of 60 mg/kg BW of pure carnosine elicited side-effects like headache and paraesthesia in 40% of the subjects. These symptoms are identical to the ones often reported following intake of high doses of β-alanine [30]. Some recent studies in our lab (manuscript in preparation) tested supplementation of combined pure carnosine and anserine, in a dose of 30 mg/kg BW of each, and did not elicit adverse symptoms. These results suggest that in contrast to high doses of pure carnosine, the combined ingestion of carnosine and anserine – especially when present in a real food matrix like a broth - can prevent the occurrence of side-effects.

The practical implications relate to a novel approach in the quest for performance improvement in sports. We supplemented with 8 mL/kg BW of CB, corresponding with an average value of 3.5 g HCDs or 46.4 mg/kg BW. This is equivalent to roughly 350-g serving of chicken breast meat, known to have a HCD content of approximately 1 g/100 g wet muscle [3, 31, 32]. This is a realistic amount to incorporate in a pre-competition dietary plan of athletes, although it seems much more feasible to deliver it in a broth than in a real meat serving. A limitation of this study lies in the fact that we cannot fully determine whether the performance enhancing effect is attributable to the presence of carnosine and anserine in the CB, or (partly) by other macro-and micronutrients, since the placebo and experimental soups were not completely identical in regard to the latter. More specifically, the meat-derived experimental soup evidently had a higher content of protein/peptides/amino acids than the vegetarian placebo soup, but literature on potential ergogenic effects of pre-exercise protein supplementation have been limited to the potential glycogen-sparing effect in prolonged exercise [33], which cannot be extrapolated to the current brief time-trial. Another limitation is that we did not study elite athletes but rather moderately trained individuals. For some ergogenic supplements, like beetroot juice/nitrate, the further translation from discovery to highly trained athletes proved difficult [34]. It remains to be established to what extent our findings can be extrapolated to well-trained athletes and to women, who were not included in the current design. In addition to sports application, the discovery of the underlying mechanism may ultimately also lead the way to more therapeutic applications. Interestingly, the beneficial effects of the ingestion of carnosine and anserine mixtures are currently investigated and demonstrated for a number of applications, such as for cognitive function in elderly [35–38].

## Conclusions

Acute pre-exercise chicken broth ingestion significantly improved the performance of a high-intensity, short-lasting cycling exercise. Circulating carnosine and anserine concentrations were markedly elevated but we currently could not pinpoint improved acid-base balance nor antioxidant function as potential ergogenic mechanisms. The full extent of the performance spectrum that benefits from CB supplementation remains to be established.

## Data Availability

The datasets used and/or analysed during the current study are available from the corresponding author on reasonable request.

## Abbreviations

ACN: Acetonitrile
BW: Body weight
CB: Chicken broth
CBEX: Chicken breast extract
CN1: Human serum carnosinase-1 enzyme
GSH: Reduced glutathione
GSSG: Oxidized glutathione
FA: Formic acid
HCD: Histidine-containing dipeptides
LOD: Limit of detection
PLA: Placebo soup
SSA: Sulfosalicylic acid
TBARS: Thiobarbituric acid reactive substances
TT: Time trial
